# Characterization of the Chemical Resistance of Two Non-Vulcanized Styrene–Butadiene Rubbers (SBR)

**DOI:** 10.3390/ma17122925

**Published:** 2024-06-14

**Authors:** Florian Kempis, Jeanette Orlowsky

**Affiliations:** Faculty of Architecture and Civil Engineering, Department of Building Materials, TU Dortmund University, August-Schmidt-Str. 8, 44227 Dortmund, Germany; jeanette.orlowsky@tu-dortmund.de

**Keywords:** alkali resistance, AR glass, durability, polymers, SBR, textile concrete

## Abstract

The objective of this series of tests is to characterize the alkali and water resistance of two non-vulcanized formulations based on co-polymerizing styrene–butadiene rubbers (SBR1 and SBR2). The relative merits of the two polymer systems as impregnating agents for alkali-resistant glass reinforcement in cementitious binders are assessed. For this purpose, polymer films were synthesized and then chemically conditioned for up to half a year at temperatures of 23 °C and 50 °C in sodium hydroxide and potassium hydroxide solutions as well as in salt and distilled water. Changes in mass, tensile strength, and material hardness were evaluated to assess the chemical resistance of the two polymer systems. The different test liquids generally led to swelling (increase in mass) and degradation (reduction in mass) of the polymer structures. These two processes occurred simultaneously. The liquid absorption capacity of the SBR1 impregnation system was between 25.05% and 51.60% by weight, depending on the test liquids. In contrast, the SBR2 impregnation system exhibited a lower liquid absorption capacity, with a weight increase from 21.19% to 42.90%. The chemical conditioning resulted in a maximum mass reduction of the polymer structure SBR1 of 8.82% by weight. The polymer SBR2, on the other hand, only lost up to 2.88% by weight. The tensile strengths of the unconditioned samples of the polymer systems SBR1 and SBR2 were 55.49 ± 7.47 N and 80.87 ± 15.96 N, respectively. The test liquids caused a reduction in strength over the storage period which was accelerated by increased temperatures. The loss of strength of the polymer structure SBR2 was lower over the entire conditioning period. In this context, a correlation was found between strength and material hardness. Overall, the polymeric impregnation system SBR2 had a lower liquid absorption capacity and a lower degree of damage caused by the degrading test liquids. Furthermore, the tensile strength was generally higher and more robust over the entire conditioning period. The results of the durability tests indicate that the SBR2 polymer system is more suitable for use in cementitious binders, as it exhibits less degradation of the polymer structure in response to chemical aging processes.

## 1. Introduction

The composite material textile-reinforced concrete has a long service life, enables the formation of a wide variety of geometries, and, in contrast to classic reinforced concrete, requires significantly less binder. In this respect, this material is becoming increasingly important in the construction sector and is gaining acceptance in society.

It is mainly carbon and alkali-resistant glass textiles (AR glass textiles) that are used in the construction industry. Technical textiles consist of synthetic fibers arranged in a grid pattern. These are usually woven or laid at right angles to each other. The fiber strands are divided into warp and weft threads. The warp direction (or 0° direction) shows the production direction. The weft direction (or 90° direction) is orthogonal to the warp direction. To connect the fiber strands, they are often fixed with a warp knitted yarn.

These technical textiles are predominantly impregnated with synthetic polymer dispersions (acrylates, styrene–butadiene) or modified epoxy resins.

In terms of sustainability, polymer-impregnated AR glass is more attractive than classic reinforced concrete or the currently more sustainable carbon textiles owing to the significantly lower carbon footprint during the manufacturing process. The synthetic polymer dispersions are much more resource-efficient to manufacture than epoxy resins, as these have to be thermally treated at temperatures of 200–3000 °C during the multi-stage manufacturing process [1]. In addition, epoxy resins pose health risks during use; they are classified as a sensitizing substance group according to the GHS label. In particular, the issue of bisphenol A, which is associated with epoxy resins and is hazardous to the environment and health, has not been sufficiently clarified. In view of the numerous controversial discussions, bisphenol A still cannot be clearly classified with regard to its carcinogenicity, genotoxicity, and reproductive toxicity for humans [2,3,4].

There is still considerable potential for further development and improvement of the performance of these impregnations.

AR glass is not completely resistant to alkaline (concrete pore) solutions. More precisely, AR glass is only more resistant in the long term than other types of glass. This fact correlates with a time-dependent deterioration of mechanical performance. Impregnations can counteract such a negative development of performance [5,6,7,8,9].

The impregnation system applied to AR glass acts as a physico-chemical protective layer. The impregnation protects the impregnated textile from mechanical damage caused by external influences and/or from (aggressive) alkaline pore solutions.

The prerequisite for this is a robust, almost diffusion-tight, and extremely resistant impregnation with regard to alkaline concrete pore solutions. As a result, an impregnation system must meet a number of general requirements to achieve effective impregnation quality.

These include a sufficiently low viscosity for optimum penetration of the individual filaments or textiles into the multifilament core as well as good wettability and adhesion to the material surface. Furthermore, the impregnated multifilament yarn or textile must have excellent cohesive strength to transfer high bonding forces. It should also have high dimensional stability under heat (up to approx. 80 °C in use) and a low tendency to creep [10].

It is therefore essential to characterize the resistance of impregnated AR glass by reference to changes in properties caused by environmental conditions over time.

Polymeric impregnation systems are exposed to a variety of physical and chemical influences, which, among other things, subject them to a gradual ageing process.

The triggers for a progressive change in polymer structure are divided into internal and external causes of ageing. The internal causes of ageing are due to thermodynamically unstable structural states of the polymer which are the result of incomplete polymer synthesis and/or are triggered by residual and orientation stresses as well as limited miscibility. The external causes of ageing are chemical–physical and exert microbiological effects on the polymer structure. These include inorganic and organic media, mechanical stresses, temperature changes, radiation, and damage or degradation by microorganisms [11,12].

A general distinction is made between physical and chemical ageing processes.

Physical ageing processes cause changes in the polymer structure, the molecular order, and the concentration ratios, all of which can be reversed by re-melting. The physical polymer structure is changed without manipulating the chemical structure of the molecular chains [12,13,14].

The chemical ageing processes, on the other hand, cause a degradation of the polymer structure which is not reversible by re-melting. The macromolecules are degraded, for example, by diffusion-controlled oxidation or by the process of hydrolysis during chemical degradation.

In addition, the mechanical properties of a polymer can change temporarily due to post-condensation and post-polymerization. As a result of these processes, the chemical composition and molecular structure are changed again.

Basically, the distinction between the effects of physical and chemical ageing processes proves to be extremely complex, as the different effects of the processes described can overlap.

The aim of this series of tests is to assess the durability of two non-vulcanized formulations based on co-polymerizing styrene–butadiene rubbers (hereinafter referred to as SBR1 and SBR2) which were exposed to external causes of ageing over a defined period of time. The thermoplastics SBR1 and SBR2 were chemically conditioned in order to determine their alkali and hydrolysis resistance from a materials science perspective. The resistance of the two polymer systems is used to assess whether they could be used as impregnating agents for alkali-resistant glass reinforcement in cementitious binders.

If the resistance of the polymer systems is above average, the loss of strength of the glass fibers in cementitious binders could be significantly delayed. If the serviceability of glass-fiber-reinforced textile concretes is in the range of classic reinforced concrete, the use of glass-fiber-reinforced concretes would be more sustainable and beneficial from an ecological and economic point of view.

## 2. Materials and Methods

### 2.1. Polymer Synthesis

**Figure 1:**
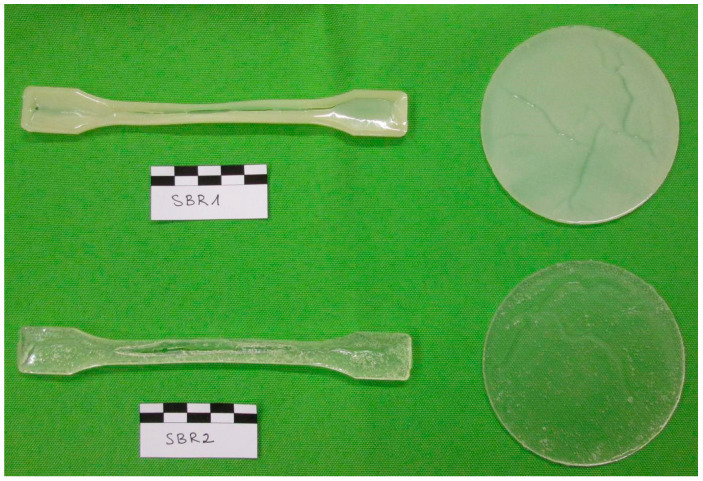
Overview image of the synthesized round and dog bone samples SBR1 and SBR2. The dog-bone samples are 170 mm long; the diameter of the round samples is 90 mm.

Polymer films (round and dog-bone samples) were synthesized in the laboratory of the Chair of Construction Materials at TU Dortmund University from two aqueous dispersions developed by CHT-Group Germany, Tübingen (Baden-Württemberg), Germany (Figure 1). The film formation of the two polymer dispersions took place in PTFE molds over a period of 24 h and at a temperature of 23 °C at 50% relative humidity. The stabilized polymer films were immediately annealed at temperatures of 120 °C for three minutes and at 150 °C for two minutes.

The stabilization process developed in preliminary tests provided the best possible results with regard to the formation of defects (inhomogeneities) during film formation. However, it was not possible to produce completely defect-free polymer films, although a large number of stabilization processes were investigated under different climatic conditions. The inhomogeneities of the respective polymer films have a similar extent, which makes it possible to compare the material characteristics with each other.

The sample geometry of the round samples with a diameter of 90 mm and a thickness between 570 µm and 1110 µm is suitable for determining the water vapor diffusion resistance number µ. In this respect, the sample format of the round polymer samples was selected according to the specifications of [15]. The determination of the water vapor permeability was not determined in this work, as this is part of a further test matrix.

The format of the dog-bone samples (type 1A) was selected in accordance with the specifications from [16].

### 2.2. Chemical Conditioning and Sample Preparation

The synthesized polymer films SBR1 and SBR2 were stored airtight for up to six months at 23 °C and 50 °C in polypropylene containers which were filled with various alkaline test liquids and distilled water (Table 1). The pH value of the test liquids was checked regularly using the pH meter CG 822 from Schott Geräte GmbH, Mainz (Rheinland-Pfalz), Germany.

The samples were mostly removed from storage after 7, 14, and 28 days (short-term conditioning) and after three months and six months (long-term conditioning), with the exception of samples for chemical conditioning for the purpose of investigating liquid absorption capacity (See Section 2.3). Following storage, the polymer films were kept in a desiccator at room temperature for 24 h until the respective materials science tests (preconditioning).

### 2.3. Liquid Absorption Capacity

The term hygrothermal effect describes the effect of different temperatures and humidity on the polymer structure. In principle, this combined effect has a greater impact on the mechanical material properties of polymers than just one of the two parameters mentioned. The moisture absorption of free polymer films is mainly controlled by diffusion processes according to Fick’s law [17]. Aqueous solutions can also be absorbed capillary into the cavity volume of the polymer structure via voids until a saturation limit is finally reached [14].

The weight gain due to liquid absorption by some polymers can be a lengthy process under certain circumstances. In this respect, the effective liquid absorption capacity of polymer structures can only be inadequately described on the basis of the moisture cycles prescribed in the currently valid normative references [18,19]. Accordingly, the round polymer samples were deliberately stored for longer in order to record the effective liquid absorption capacity up to the actual saturation limit. The synthesized round polymer samples SBR1 and SBR2 were dried at 50 °C until constant mass before storage according to the specifications of [19]. Depending on the sample thickness, this process took up to two days. In the next step, the polymer films were placed in PP wide-neck bottles, which were then filled with the various test liquids and sealed. The round polymer films in the containers were then stored in two drying cabinets at temperatures of 23 °C and 50 °C.

The amount of liquid absorbed by the polymer structure was determined based on the change in mass according to [19]. The relative (percentage) mass change c*_p_* is defined by the difference between the input mass m*_t_* (weight at the time of removal from storage) and the output mass m*_const._* (weight at constant mass). Accordingly, the change in mass c*_p_* at the respective removal times is calculated using the following equation:

(1) $$c_{p}=\frac{m_{t}-m_{\mathrm{const}.}}{m_{\mathrm{const}.}}\times 100$$

The changes in mass of round polymer films are given as the arithmetic mean of three individual weighings, which were determined after 24, 48, 72, and 96 h as well as 7 and 28 days and after three, four, five, and six months.

### 2.4. Thermogravimetric Analysis (TGA)

The chemical reactions between the diffusing molecules of the test liquids and the polymer systems SBR1 and SBR2 cause changes in the polymer structures. The samples stored in the various test liquids were mainly subject to diffusion-controlled chemical ageing processes, which can cause the degradation of the polymer network and/or the temporary formation of new network nodes. In principle, these two effects can work in parallel.

Depending on the structural properties and the chemical conditioning regime, the influence of one of the two effects is always more dominant [20]. The thermogravimetric analysis of samples of the chemically conditioned polymer systems SBR1 and SBR2 allows conclusions to be drawn about the possible extraction of water-soluble and non-alkali-resistant components from the polymer structures (degradation). Furthermore, the decomposition processes of the impregnation systems can be characterized at higher temperatures and volatile ingredients can be detected.

Depending on a changing heating rate, the mass changes of up to five individual samples per conditioning regime were analyzed using the TGA/DSC3+ from Mettler Toledo. At the beginning of the method, the samples were heated to 105 °C at a heating rate of 20 K/min to evaporate free water in the cavity volume of the polymer structure. The temperature of 105 °C was maintained for 10 min. According to a representative number of preliminary tests with the chemically conditioned polymers, no change in mass could be detected after about 7 min.

In the next step, a temperature of 250 °C was reached at a heating rate of 20 K/min. This temperature was also maintained for 10 min to remove volatile components (solvents, monomers, test liquids).

In the final step, the samples were heated at a heating rate of 10 K/min to a temperature of 650 °C, where the polymer structures began to melt and then decompose almost completely. The samples were annealed at a final temperature of 650 °C for 10 min. Higher temperatures did not lead to deviating mass changes. The change in mass of the conditioned samples was evaluated according to [21] using the STARe Excellence software (version 16.30) from Mettler Toledo, Greifensee (Zurich), Switzerland (one-step mass changes).

### 2.5. Determining the Tensile Strength (Dog Bone Tensile Test)

The polymer samples were tested using uniaxial film tensile tests based on [22] in accordance with the specifications of [16] at room temperature until material failure.

**Figure 2:**
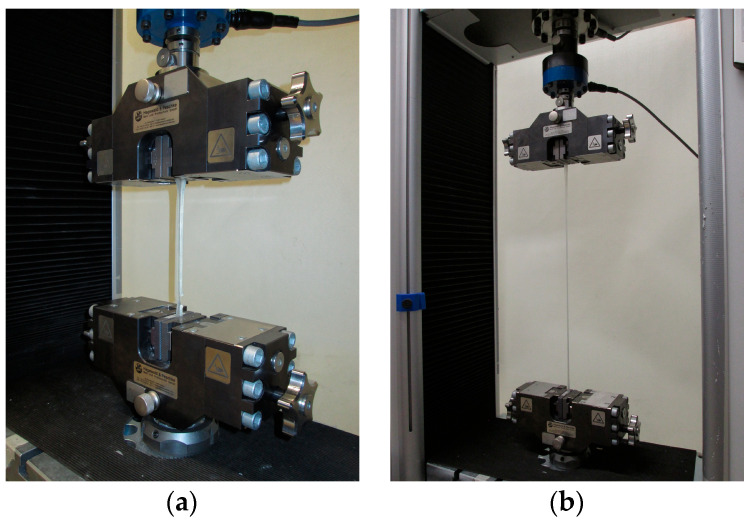
(**a**) Clamped dog-bone sample before testing; (**b**) testing the tensile strength of a dog-bone sample.

The film tensile tests were carried out using an Inspekt 100 kN universal testing machine from Hegewald und Peschke Mess- und Prüftechnik GmbH, Nossen (Sachsen), Germany (Figure 2). The sample and ambient temperature have a major influence on the strength of the polymer structures, which is why the tests were carried out under almost identical climatic conditions [23,24]. The room temperature was controlled before the start of the test and was maintained in the range of 18 °C to 23 °C. A total of up to ten individual samples were tested for each conditioning scenario.

Before the film tensile tests, a tightening torque of 12.5 N was defined for the contact pressure of the prepared specimens between the clamping jaws of the two wedge screw grips. The dog bones mounted using wedge screw grips were fixed with profiled metal clamping jaws. This method of installation has proven to be expedient, firstly because the specimens largely fail in the area of the free path length and, secondly, because the polymers are not pulled out of the metal clamping jaws under load.

The approach and test speeds during the film tensile tests were set extremely high compared to tests on other building materials such as concrete or steel, as the polymer dog bones are extremely ductile. Due to this material property, the test speed can influence the strength and elongation of the polymer systems. This fact applies in particular to viscoelastic materials such as the styrene–butadiene rubbers studied in this series of tests. The strength of the polymer dog-bone samples was only compared with each other, which is why the test conditions were defined at our own discretion.

The start-up speed up to the preload force of 5 N was 200 mm/min. The test speed was reduced to 100 mm/min once the preload force was reached. The test ended as soon as a force drop of 90% was detected. The test parameters mentioned proved to be appropriate with regard to the test sequence in preliminary tests. As the specimen cross-section is not constant over the specimen length due to the manufacturing process (Figure 1), the calculation of strength is omitted in the evaluation and only the tear force is discussed.

### 2.6. Shore Hardness A

In addition to tensile strength, hardness is another important material parameter for characterizing a polymer system, especially when used as an impregnating agent in cementitious binders, as it contributes significantly to dimensional stability and bonding properties.

In this work, the hardness of the polymer systems was determined on the waisted dog-bone samples using a Shore A durometer according to [25] shortly before the tensile strength was determined on the dog-bone samples. The dog-bone samples were approximately 4 mm to 5 mm thick in the edge area, so that the test specimens met the normative requirements. However, the dog-bone samples did not have an ideal rectangular cross-section. As a result, the test was carried out along the stiff edge areas of the dog-bone samples to minimize possible falsification of the measurement results due to the varying cross-section. The polymer dog-bone samples were placed with the smooth side on a stable and deformation-resistant base. Each specimen was then tested at least three times.

## 3. Results and Discussions

In principle, it can be seen that all chemical ageing processes followed the Arrhenius relation. The consequences of some effects are explained by way of example at lower or higher temperatures.

### 3.1. Liquid Absorption Capacity

**Figure 3:**
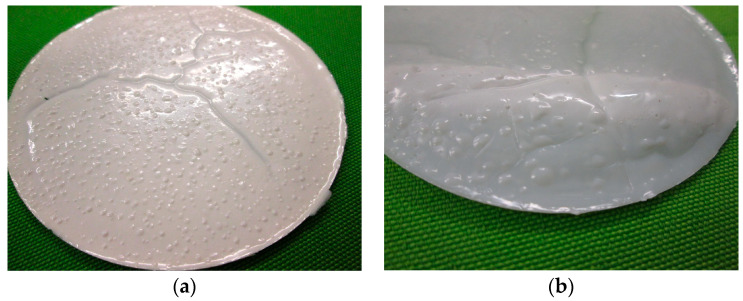
(**a**) SBR1_NaCl_ after 7 days at 23 °C; (**b**) SBR2_H2O_ after 7 days at 23 °C.

Due to the wide-meshed (thermoplastic) polymer structure of the round samples, liquid absorption was significantly faster at the beginning of storage than toward the end of the conditioning period. The result from liquid absorption by diffusion, capillary action, and transport along microcracks was swelling and volume change in all polymer films, which were superimposed by extraction processes (Figure 3). As a result, the actual weight and volume change was a balance of liquid absorption (weight increase) and dissolution processes (weight decrease). Furthermore, the test liquids had a softening, physical effect on the round polymer samples, which, however, hardened again a few hours after storage.

The round polymer films SBR1_NaOH, KOH_ stored in sodium hydroxide and potassium hydroxide solution already had a significant weight increase of 20% by weight on average after 24 h at a storage temperature of 23 °C. In contrast, the weight gain of the samples stored in salt water and distilled water was significantly lower at around 5% by weight. Basically, the polymer structure showed a continuous increase in mass over a period of 4380 h. It is noteworthy that the samples stored in salt water continuously showed a significantly lower increase in weight than the other round polymer films. The round films stored in distilled water generally showed a slower increase in mass which, toward the end of conditioning, was at the level of the samples conditioned in alkaline media. The supposed capacity of the polymer round films was reached between 2190 h and 3650 h.

**Figure 4:**
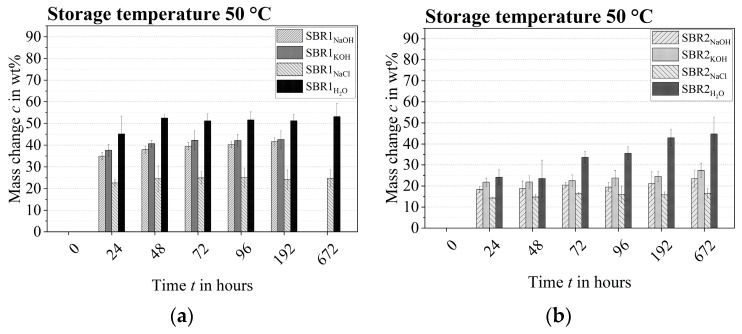
Change in mass of the round polymer samples at 50 °C at the respective ageing times: (**a**) SBR1, (**b**) SBR2.

The ratio of the weight increases of the chemically conditioned polymer films at 50 °C were equivalent to those of the samples stored at 23 °C. However, the higher-temperature samples already showed such a large weight increase after 24 h, only achieved toward the end of the durability test at a storage temperature of 23 °C. The actual capacity or saturation limit of the polymer structure SBR1 was reached between 72 h and 96 h (Figure 4a). The polymer films stored in sodium hydroxide and potassium hydroxide solution could no longer be weighed accurately after 672 h, as the polymer structure had begun to decompose. This onset of decomposition could already be recognized macroscopically after about 200 h. In contrast, the samples conditioned in salt and distilled water only gradually dissolved after approximately 2000 h.

The development of the increase in weight of the SBR2 polymer round films chemically conditioned at 23 °C was largely analogous to the changes in mass of the SBR1 samples. The salt water absorption capacity of the polymer structure SBR2 was also conspicuously low compared to the other test liquids. The exception, however, was the absorption capacity for distilled water which was significantly higher in the SBR2 polymer films right from the start.

The weight increase of the polymer structure stored in sodium hydroxide and potassium hydroxide solution had an almost identical capacity which only changed after some time. The water absorption capacity of the polymer structure favored a continuous increase in mass from about 192 h, while the other round polymer films had already reached their supposed saturation limit after 192 h.

The salt water and water absorption capacity of the polymer films SBR1 and SBR2 was almost identical at a storage temperature of 23 °C between 3650 h and 4380 h. In contrast, the liquid absorption of sodium hydroxide and potassium hydroxide solution was noticeably lower.

In the same way as with the round polymer films SBR1, the weight increases of the chemically conditioned polymer films SBR2 tended to be identical at higher temperatures. According to the Arrhenius relation, liquid absorption was accelerated at a temperature of 50 °C. The actual saturation limit was reached between 96 h and 192 h.

The SBR2 round films stored in sodium hydroxide and potassium hydroxide solution showed no signs of decomposition after 672 h, which is why they could be weighed without any problems (Figure 4b). The dissolution of the SBR2 polymer structure in the highly alkaline solutions only began after around 1800 h. In contrast, the polymer films exposed to salt water and distilled water were almost macroscopically intact even after 4380 h.

In summary, the polymer structure SBR1 had a significantly higher liquid absorption capacity than the polymer structure SBR2 (Table 2). This, in turn, explains, among other things, the better alkali and hydrolysis resistance of the SBR2 polymer round films.

### 3.2. TGA

The onset temperatures of both impregnation systems were within a similar temperature range, regardless of the conditioning regimes. The melting process of the polymer structures began in a temperature range of 383.38 ± 2.21 °C to 386.63 ± 1.38 °C. The decomposition of the polymers was completed between 473.30 ± 0.36 and 474.28 ± 0.91. The total mass loss *m*_F_ of the polymer systems was almost identical (Table 3).

The liquid absorption capacity of the cavity volume (swelling capacity) in conjunction with superimposed diffusion and dissolution processes had an effect on the changes in mass of the chemically conditioned polymer structures. If the extent of the dissolution processes was higher than the swelling capacity, this inevitably led to a decrease in volume or mass. This mechanism worked until a time- and temperature-dependent equilibrium was reached. The volume or mass was below the reference value when the equilibrium was reached. It is also possible that polymer structures experienced a continuous increase in volume or mass due to oxidation caused by certain media.

**Figure 5:**
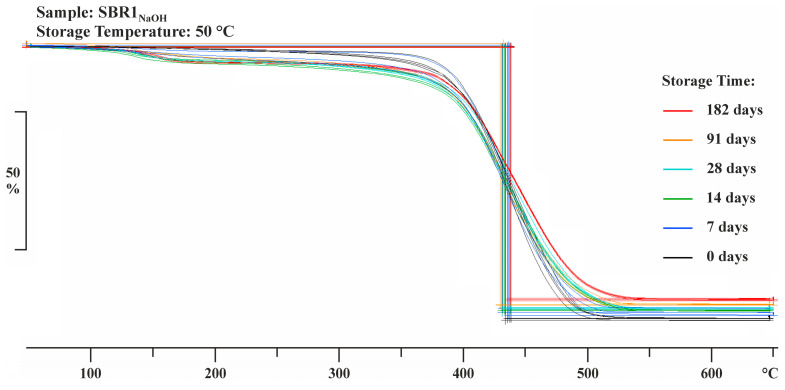
TGA measurement curves of the polymer films SBR1 stored in sodium hydroxide solution at 50 °C for up to six months. The changes in mass were evaluated using the STARe Excellence software from Mettler Toledo (step evaluation).

**Figure 6:**
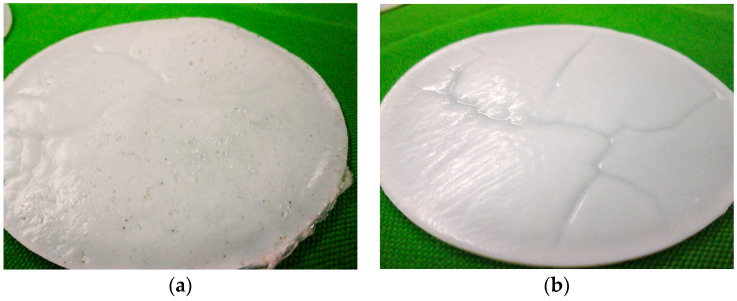
(**a**) Moderately degraded surface structure of a round polymer film SBR1 stored for 14 days at 23 °C in sodium hydroxide solution. (**b**) Largely unchanged surface structure of a round polymer film SBR2 stored for 14 days at 23 °C in sodium hydroxide solution.

The changes in the mass of the polymer samples varied depending on the conditioning regimes and, for the most part, had a lower overall mass loss after exposure, a phenomenon which basically indicates the decomposition of the polymer structures (exemplary Figure 5 and Figure 6)

The exception was the group of samples stored in salt water which showed a marginal increase in mass compared to the reference sample at the beginning of conditioning and only a minimal reduction in weight toward the end of storage. This development can almost certainly be explained by the fact that salt crystals were still present on or possibly within the cavity volume of the polymer structure after their removal from storage and preconditioning.

The lowest total mass loss was observed in the polymer films SBR1 stored in distilled water, an indication of molar mass degradation due to the cleavage of hydrolysable groups from the main chain of the polymer structure.

**Figure 7:**
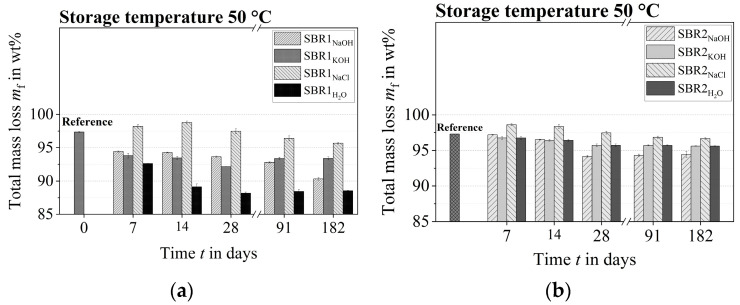
Mass loss of the polymer samples at 50 °C at the respective ageing times: (**a**) SBR1, (**b**) SBR2.

A side effect of hydrolysis was the embrittlement of the polymer structure due to the shortening of the main chain [26]. This was also observed after removal of the polymer films SBR1. In addition, the polymer films stored in sodium hydroxide and potassium hydroxide solution also clearly showed lower total mass losses compared to the reference, which led to changes in the molecular structure, formation of functional groups, or the cleavage of low molecular weight products. The test liquids caused an overall degradation of the polymer structure SBR1 which was significantly accelerated by an increase in temperature (Figure 7a).

The aqueous solutions also led to mass changes in the polymer samples SBR2 which can be attributed to a degradation of the polymer structure. However, this degradation was much less pronounced. The mass loss of the polymer structure SBR2 was basically unchanged between 28 days and 6 months in the 50 °C tempered media (Figure 7b).

The dissolution processes outweighed the swelling capacity of the polymer systems SBR1 and SBR2, leading to an overall decrease in mass (especially at higher temperatures). Although the round polymer films stored in salt water initially showed an increase in mass, a longer conditioning period at higher temperatures nevertheless led to a degradation of the polymer structures.

Accordingly, the polymer structure SBR2 had better alkali and hydrolysis resistance than the polymer structure SBR1. Particularly at a storage temperature of 23 °C, the differently conditioned polymer samples SBR2 only showed noticeable changes in the total mass loss after six months, while noticeable mass changes were measured in the polymer samples SBR1 after just seven days.

### 3.3. Dog Bone Tensile Test

**Figure 8:**
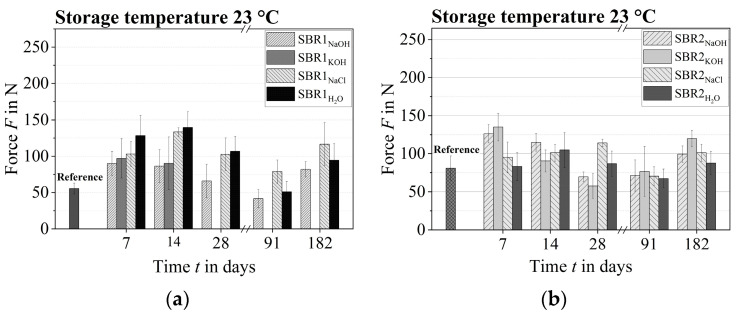
Tearing forces of the dog-bone samples at 23 °C at the respective ageing times: (**a**) SBR1, (**b**) SBR2.

The tearing forces of the two polymer systems SBR1 and SBR2 increased over a period of 7 to 14 days at a storage temperature of 23 °C (Figure 8). One possible explanation for this is the formation of new cross-links within the polymer structure during chemical ageing and the resulting higher cross-link density. The technical literature maintains that the cross-linking density of styrene–butadiene rubbers can increase in the initial phase of (artificial) weathering and decrease again later on. In general, an increase in the degree of cross-linking correlates with an increase in the strength and hardness of the polymer structure [27].

At this point, it must be pointed out that the resistance of vulcanized styrene–butadiene rubbers (elastomers) was primarily investigated in the specialist literature. Further studies on the chemical resistance of non-vulcanized styrene–butadiene rubbers (thermoplastics), as in the test series presented here, have not been carried out.

A reduction in the tensile strength of the dog-bone samples at 23 °C was first observed after 28 days. However, at the end of the short-time conditioning, the tensile strengths of some of the samples stored in salt water and distilled water (taking into account the error interval) were still above the reference level of 55.49 ± 7.46 N. In contrast, the SBR1 dog-bone sample exposed to sodium hydroxide and potassium hydroxide solution showed a noticeable loss of strength. The SBR1 samples could no longer be tested after 28 days, as the polymer structure had essentially decomposed. In contrast, the strength of the SBR2 samples stored in sodium hydroxide and potassium hydroxide solution at 23 °C could be tested at any time. Over the entire period, no serious reduction in the strength of the SBR2 polymer system was observed.

The tearing forces of the polymer systems SBR1 and SBR2 of the other samples decreased after a quarter of a year to the approximate level of the unconditioned reference samples.

Unexpectedly, there appeared to be a further increase in strength after six months. Such an increase in strength could possibly be due to a crack-like and hardened surface texture of the polymer films. This media-independent surface effect could be observed between five months and six months. However, a possible increase should be classified as rather unlikely, taking into account the measured value scatter.

The SBR1 dog-bone samples stored in sodium hydroxide and potassium hydroxide solution at 50 °C were no longer testable after just seven days, as the polymer structure had largely decomposed. In contrast, the strength of the SBR2 samples could be measured without any problems even after 14 days. Only the SBR2 samples conditioned in potassium hydroxide solution were so degraded after 28 days that a strength test could not be carried out. Equivalent to the lower-temperature samples, an increase in strength (in the testable dog-bone samples) was observed at the beginning of conditioning at 50 °C.

Essentially, all dog-bone samples of the two polymer systems had been intensively damaged by the different test liquids between two months and six months at 50 °C, which is why no measured values were generated.

### 3.4. Shore Hardness A

**Figure 9:**
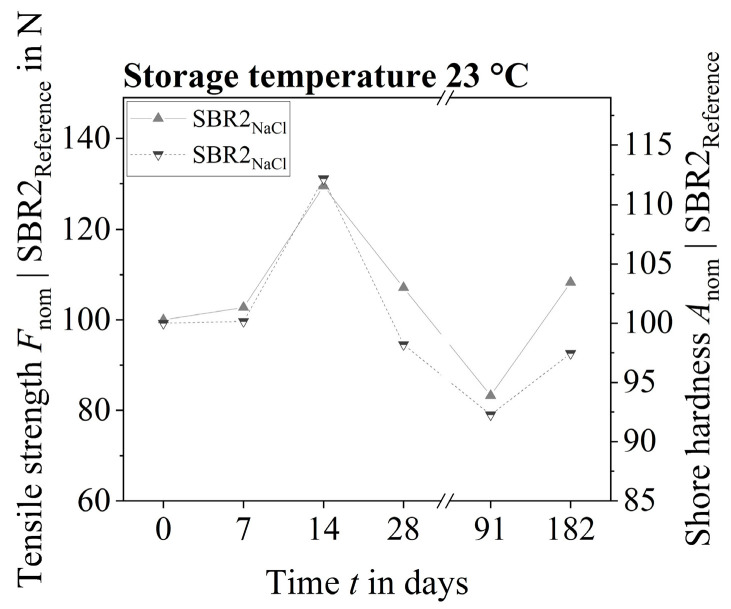
Correlation of the tensile strength *F*_nom_ and the Shore hardness *A*_nom_ nominated to the respective (non-chemically conditioned) reference values SBR2_Reference_.

In principle, the measured values of Shore hardness A correlated with the measured values of tensile strength N_max_ over the entire storage period. The correlation of these material parameters could be determined for all polymer samples that were stored at a temperature of 23 °C (cf. [28]). The standardization of the chemically conditioned samples to the non-stored reference samples showed that the measured values of tensile strength and Shore hardness correlated over time (Figure 9).

**Figure 10:**
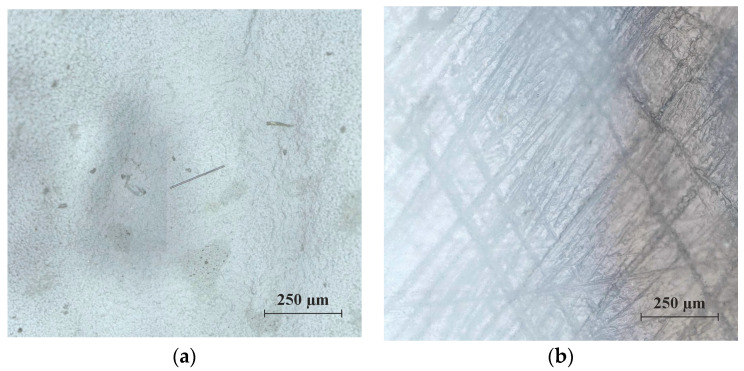
(**a**) Microscopic image of the surface texture of an unconditioned reference sample of the SBR2 polymer system, (**b**) microscopic image of the brittle, cracked surface effect of a sample of the SBR2 polymer system stored for six months in distilled water. The images were taken with the Axio Imager.M2m research microscope from Carl Zeiss, Oberkochen (Baden-Württemberg), Germany. Objective used: EC objectives (Epiplan-Apochromat series), magnification: 20×.

In the initial phase of this chemical conditioning, the aqueous media led to an increase in hardness of between 7 days and 14 days. The polymer structures then softened due to chemical ageing processes, which is why a significant reduction in hardness could be observed after three months. Equivalent to a possible increase in tensile strength toward the end of the storage period, the hardness also increased (Figure 10a as an example).

Characteristic of the polymer films examined was the surface cracking toward the end of the chemical conditioning, leading to embrittlement of the polymer structures (Figure 10b). This surface effect—which could possibly also be responsible for an increase in strength—led to an increase in the material hardness of the polymer systems. The changes due to chemical conditioning were less “fluctuating” in the SBR2 polymer system.

The described surface effect was not observed at the beginning of chemical conditioning.

## 4. Conclusions

The investigated conditioning had a detrimental effect on the alkali and hydrolysis resistance of the SBR1 and SBR2 polymer systems by initiating or accelerating physical and chemical ageing processes.

Regardless of the test liquid, the storage temperature of 23 °C caused an increase in the tensile strength of the two polymer impregnation systems SBR1 and SBR2 in a period of 7 days to 14 days. A reduction in tensile strength was first measured after 28 days. The dog-bone samples could be tested without any problems until the end of the conditioning period, as the polymer structure did not decompose significantly. On the other hand, the storage temperature of 50 °C caused early decomposition of the SBR1 and SBR2 polymer systems, which is why most of the dog-bone samples could not be tested without defects.

There was a correlation between the change in tensile strength and hardness of the polymer structures over the storage period. The increases in strength observed at the start of the chemical conditioning covaried with the hardness of the material (Shore hardness A). The temporary increase in strength is an indication that the polymerization reactions had not been fully completed. Consequently, the polymer synthesis should be optimized to reduce the reduction of residual stresses and increase the density of the polymer structures. Accordingly, the technical performance of the two polymer systems has not yet been fully exploited.

Furthermore, swelling led to changes in the polymer structures, on the one hand, and dissolution processes led to the degradation of the polymer structures, on the other. In contrast to the SBR2 polymer system, the SBR1 polymer system had a significantly higher liquid absorption capacity and overall lower mass loss, as evidenced by the more intense chemical degradation and change in polymer structure. The actual capacity of the SBR1 impregnation system was between 25.05% and 51.60% by weight, depending on the test liquids. The SBR2 impregnation system, on the other hand, had a lower actual capacity with a weight increase of 21.19% to 42.90% by weight.

The reduction in mass due to the degradation of the polymer systems influenced the increase in weight due to the test liquid absorption capacity of the polymers. The polymer structure SBR1 lost between 1.05% and 3.74% by weight at a storage temperature of 23 °C, while the mass of the SBR2 polymer system was reduced by a maximum of 1.81% by weight. An increase in the storage temperature to 50 °C resulted in a reduction of the mass of the polymer structure SBR1 of up to 8.82% by weight. In contrast, the mass loss of SBR2 was clearly lower at a maximum of 2.88% by weight.

In conclusion, this means that the SBR2 impregnation system exhibited a reduced liquid absorption capacity and a diminished degree of damage caused by the degrading test liquids. These physical and chemical mechanisms were accelerated by higher temperatures, resulting in the premature decomposition of the polymer structure SBR1 stored in sodium hydroxide and potassium hydroxide solutions. In contrast, the decomposition of the SBR2 polymer system commenced at a later stage.

Consequently, the SBR2 polymer system as a whole exhibited markedly superior chemical resistance. The actual capacity and the maximum mass loss of the SBR2 polymer impregnation system are notably lower. Furthermore, the tensile strength of 80.87 ± 15.96 N is demonstrably higher than that of SBR1 at 55.49 ± 7.47 N and is more robust over the entire conditioning period.

For these reasons, it can initially be assumed that the SBR2 polymer system is more suitable for use as an impregnating agent for glass fibers.

However, this statement must be verified in more detail from a materials science perspective, as other physical variables become more important during the impregnation process of glass fibers (multifilament yarns).

The surface tension and viscosity of the two aqueous polymer dispersions are decisive for the impregnation quality of multifilament yarns, scrims, or fabrics. It is conceivable that the aqueous polymer dispersion SBR1, for example, may result in a more complete wetting of individual filaments of the textiles owing to a more suitable surface tension and lower viscosity. This could, in turn, enhance the performance of the polymer system in cementitious binders, as a greater number of filaments are involved in load transfer. In this context, it is necessary to investigate the heat resistance and creep tendency of the two polymer systems, as these material parameters also have an effect on the serviceability of textile concretes.

## Figures and Tables

**Table 1:** List of the test liquids used in the investigations.

Test Liquid	pH Value
2.5 percent sodium hydroxide solution	13.4
2.5 percent potassium hydroxide solution	13.6
3.0 percent natrium chloride solution	9.1
distilled water	7.0

**Table 2:** Change in mass of the SBR2 polymers at 23 °C and 50 °C after 4380 h and 672 h, respectively.

Test Liquid	Storage Temperature 23 °C *SBR2* in wt%	Storage Temperature 50 °C *SBR2* in wt%
NaOH	17.0 ± 3.5	23.4 ± 4.1
KOH	20.5 ± 4.5	27.35 ± 3.4
NaCl	14.0 ± 2.5	16.5 ± 2.3
H_2_O	43.9 ± 3.4	56.8 ± 7.9

**Table 3:** Total mass loss of the polymer systems SBR1 and SBR2.

Polymer System	Total Mass Loss *m*_F_ in wt%
SBR1	97.359 ± 0.064
SBR2	97.317 ± 0.015

## Data Availability

Data are contained within the article.
